# Modulated stimuli demonstrate asymmetric interactions between hearing and vision

**DOI:** 10.1038/s41598-019-44079-5

**Published:** 2019-05-20

**Authors:** Quoc C. Vuong, Mark Laing, Anjana Prabhu, Hei Iong Tung, Adrian Rees

**Affiliations:** Institute of Neuroscience, Newcastle University, Newcastle upon Tyne, NE2 4HH UK

**Keywords:** Sensory processing, Human behaviour

## Abstract

The nature of interactions between the senses is a topic of intense interest in neuroscience, but an unresolved question is how sensory information from hearing and vision are combined when the two senses interact. A problem for testing auditory-visual interactions is devising stimuli and tasks that are equivalent in both modalities. Here we report a novel paradigm in which we first equated the discriminability of the stimuli in each modality, then tested how a distractor in the other modality affected performance. Participants discriminated pairs of amplitude-modulated tones or size-modulated visual objects in the form of a cuboid shape, alone or when a similarly modulated distractor stimulus of the other modality occurred with one of the pair. Discrimination of sound modulation depth was affected by a modulated cuboid only when their modulation rates were the same. In contrast, discrimination of cuboid modulation depth was little affected by an equivalently modulated sound. Our results suggest that what observers perceive when auditory and visual signals interact is not simply determined by the discriminability of the individual sensory inputs, but also by factors that increase the perceptual binding of these inputs, such as temporal synchrony.

## Introduction

Our senses have evolved to detect different stimulus energies, but they seldom work in isolation. Interactions between hearing and vision are readily apparent from multisensory illusions that dramatically change the location or content of sounds^1,2^, and conversations are easier to follow when we can see the speaker’s lips^3^. To understand multisensory processing we need to discover the rules by which different modalities like hearing and vision interact to generate a unified percept.

A controversial issue is how auditory and visual signals are weighted when they are combined in the brain. Some studies report vision dominates hearing^4–6^. Others, however, suggest dominance is task specific depending on which modality has greater sensitivity for the task; vision dominates for judgement of spatial location, while hearing dominates in temporal tasks requiring the detection of rapidly changing stimuli^7–10^. An extension of the latter view is the Bayesian approach that argues a modality’s influence on the multisensory interaction is weighted according to the *reliability* (inversely proportional to the variability) of the information that modality contributes to the bimodal estimate of a stimulus property^11–13^.

Attempts to define how auditory-visual signals are weighted when they interact have been further complicated by the wide variety of different stimulus types that have been employed in auditory-visual studies, from formless, flashes, or sound bursts^4,8,14^, to higher-level stimuli composed of real world objects and sounds^2,3,15^. Such transient stimuli do not reflect the kind of auditory-visual events lasting a few seconds or more that people usually experience in the real world. Consequently, the way participants respond to these widely differing stimuli may reflect the different strategies needed to perform the task in hand. Conversely, semantically meaningful, higher-level stimuli like everyday objects and speech are problematic because they may recruit post-perceptual cognitive processes and memory. Overall it remains unclear whether previous results are related to auditory-visual interactions giving rise to a unified percept or to post-perceptual (e.g., decisional) processes^16^.

To disentangle these issues, we developed a novel paradigm in which participants discriminated pairs of amplitude-modulated tones or size-modulated visual cuboid  shapes, alone, or when a similarly or differently modulated stimulus in the other modality occurred with one of the pair. Importantly, the information content relevant for the tasks in these stimuli was the same (modulation depth) in both modalities, and we equated their discriminability and variance in performance between participants. We found discrimination of the auditory stimulus was influenced by a cuboid modulated at the same rate, but discrimination of the visual stimulus was much less affected by a modulated sound. The effect of the visual distractor on auditory discrimination was more than four times the effect of the auditory distractor on visual discrimination. The dominance of vision over hearing in this study, even when stimuli are equally discriminable, suggests that multimodal interactions depend on how readily two signals are perceptually bound.

## Results

### Modulated visual shapes influence the discrimination of modulated sound

**Figure 1:**
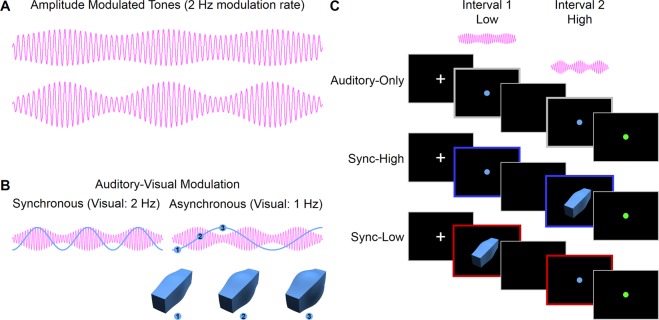
Stimuli and experimental design. (**A**) Auditory stimuli consisted of amplitude-modulated tones. A 250-Hz carrier frequency (represented in the figure by a lower frequency for clarity) was amplitude modulated at 2 Hz. Tone duration was 1500 ms so that participants heard three cycles of modulation. The lowest (20% upper) and highest (52% lower) modulation depths used in the auditory discrimination task are shown (Experiments 1 and 2). (**B**) Auditory-visual stimuli consisted of an amplitude-modulated tone as in Fig. 1A presented simultaneously with a size-modulated cuboid. In the synchronous condition (left) used in Experiment 1, the tone and cuboid had the same modulation rate (2 Hz). In an asynchronous condition (right), a condition used in Experiment 2, the tone was modulated at 2 Hz and the cuboid at 1 Hz. Numbers on the sinusoid correspond to the cuboids illustrated below which denote the object at different points in its modulation cycle (see Supplementary Videos). (**C**) The experimental paradigm. In Experiment 1, participants judged whether the modulation depth of a pair of tones was the same or different. On each trial, a 500-ms white fixation cross was presented, followed by two 1500-ms intervals separated by a 1000-ms blank screen, each containing a tone. A response was required when a green fixation dot appeared. The three different conditions are illustrated for the case in which the tone with the lower modulation depth was presented in Interval 1 and the tone with the higher modulation depth was presented in Interval 2 (i.e., a “different” trial). In the Auditory-Only condition (top), the tone in each interval was paired with a blue fixation dot. In the Sync-High condition (middle), the tone with higher modulation depth was paired with a size-modulated cuboid (70% modulation depth, Interval 2), whereas the tone with the lower modulation depth was paired with a blue fixation dot (Interval 1). In the Sync-Low condition (bottom), this pairing was reversed: the tone with the lower modulation depth was paired with the cuboid (Interval 1) and the tone with the higher modulation depth was paired with a blue fixation dot (Interval 2). In Experiment 2, two additional Async conditions were introduced (Async-High and Async-Low) in which the modulation rates of the tones and the cuboids were different (Fig. 1B right). Experiment 3 (not shown) used an analogous paradigm in which size-modulated cuboids were substituted for the amplitude-modulated tones in both stimulus intervals. In the Sync-High and Sync-Low conditions, an amplitude-modulated tone (70% modulation depth) accompanied the visual stimulus in the interval containing the more or less size-modulated cuboid, respectively.

In Experiment 1, we tested whether performance on an auditory discrimination task is affected by visual information (see Methods). We hypothesized that hearing and vision interact when information in these two modalities changes similarly, as when it derives from the same object. Such interactions lead to both modalities influencing perception, even if the information from one of the modalities does not contribute to the task in hand. Figure 1(A–C) illustrates the stimuli and paradigm used. The auditory stimuli were 250-Hz tone carriers in which the amplitude was sinusoidally modulated at 2 Hz (Fig. 1A). The visual stimulus was an arbitrary 3-D cuboid in which the size of the central portion was also sinusoidally modulated at 2 Hz (Fig. 1B left) (see Supplementary Videos). This rate is similar to the rates that predominate in the modulation envelope of continuous speech^17,18^.

Participants were tested in a sound-attenuating room using the procedure illustrated in Fig. 1C. They first completed one block of auditory-only trials, followed by one block of auditory-visual trials. In the Auditory-Only condition (Fig. 1C top), participants were presented on each trial with a pair of amplitude-modulated tones over headphones accompanied by a central blue fixation dot on the screen. They reported whether the modulation depth of the two tones was the same or different when a central green fixation dot was presented. On “different” trials one tone had a fixed modulation depth of 20% and the other had a modulation depth of either 28%, 36%, 44% or 52%; i.e., modulation depth differences of 8% to 32% in 8% steps. The fixed modulation depth occurred equally often on either interval. On “same” trials (i.e., 0% modulation depth difference), we randomly selected one of the modulation depths (20% to 52%) with replacement and presented a tone with that modulation depth in both intervals.

On auditory-visual trials, the tone in one of the intervals was paired with a cuboid whose size varied with a modulation depth of 70% in synchrony with the amplitude modulation waveform of the tone. Although the size-modulation of the cuboid was clearly visible, it contained no information relevant to the *auditory* discrimination task. In the other interval participants saw a blue fixation dot. There were two randomly interleaved pair-type conditions. In the Sync-High condition, the cuboid was presented in the interval containing the tone with the higher modulation depth (Fig. 1C middle). In the Sync-Low condition, the cuboid was presented in the interval containing the tone with the lower modulation depth (Fig. 1C bottom). On “same” trials, the cuboid was presented equally often in either interval. In this and subsequent experiments, some participants found the discrimination task difficult so we set criteria based on performance in the single-modality condition for inclusion in the analyses (see Methods).

We reasoned that if an auditory-visual interaction enhances participants’ perceived modulation depth of the paired tone, then pairing the cuboid with the tone with the higher modulation depth (Sync-High condition) should enhance the perceived difference in modulation depth between the tones in the two intervals. In contrast, when the cuboid is paired with the tone with the lower modulation depth (Sync-Low condition), its perceived modulation depth should be enhanced making it appear more similar to the more deeply modulated tone. Thus, we expected more “different” responses in the Sync-High compared to the Sync-Low condition.

**Figure 2:**
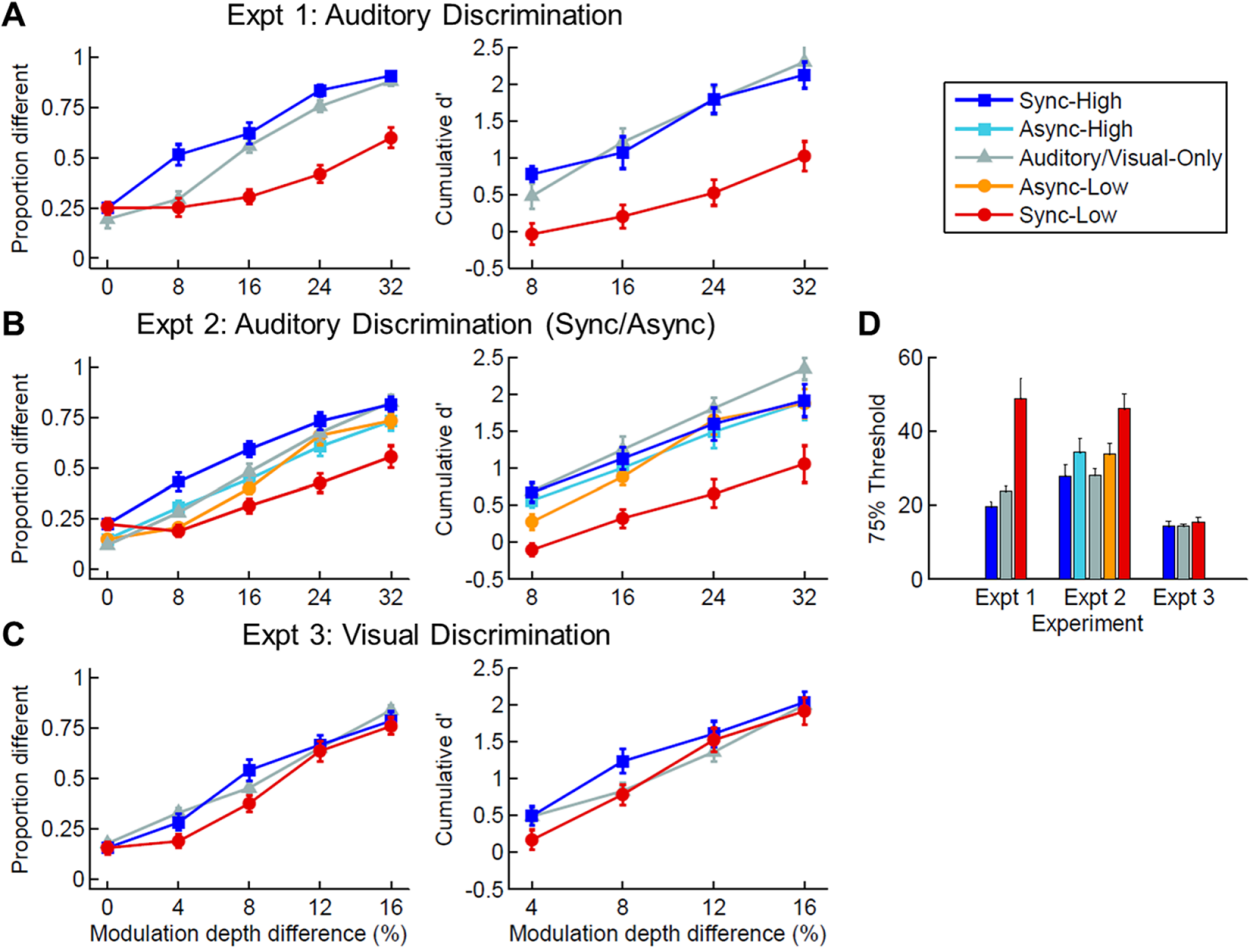
Group data (mean ± *SE*) for Experiments 1–3. (**A**) The mean proportion “different” response (left) and cumulative d’ (right) as a function of condition and modulation depth difference in Experiment 1. In the Auditory-Only condition (grey) a blue fixation dot was paired with both tones, and in the Sync-High (blue) and Sync-Low (red) conditions a modulated cuboid was paired with the tone with the higher or lower modulation depth, respectively. The modulation depth difference is the percentage difference between the modulation depth of the two tones (0%, same). (**B**) The mean proportion “different” response (left) and cumulative d’ (right) as a function of condition and modulation depth difference in Experiment 2. In the Auditory-Only condition (grey), a blue fixation dot was paired with both tones. In the Sync-High (blue) and Sync-Low (red) conditions, a modulated cuboid is synchronously paired with the tone with the higher or lower modulation depth, respectively (i.e., at the same modulation rate). In the Async-High (cyan) and Async-Low (orange) conditions, the modulation of the paired cuboid was asynchronous with that of the tone (i.e., their modulation rates were different). (**C**) The mean proportion “different” response (left) and cumulative d’ (right) as a function of condition and modulation depth difference in Experiment 3. In the Visual-Only condition (grey), both cuboids were presented in silence. In the Sync-High (blue) and Sync-Low (red) conditions, a modulated tone was paired with the cuboid with the higher or lower modulation depth, respectively. Note that sensitivity in the single modality condition across the three experiments did not differ (Experiment 1: *M* = 1.44, *SE* = 0.15; Experiment 2: *M* = 1.52, *SE* = 0.12; Experiment 3: *M* = 1.17, *SE* = 0.15). (**D)** Mean 75% threshold in Experiments 1–3 for the Sync-High (blue), Async-High (cyan), Auditory or Visual-Only (grey), Async-Low (orange) and Async-High (red) conditions. The threshold is the difference in modulation depth (percent) required for 75% discrimination performance per condition. This threshold was computed by fitting a cumulative Gaussian function to the proportion “different” data for each participant and each condition, and then estimating the modulation depth difference from the fitted curve that led to a 75% performance level. In cases in which a fit was not possible, the participant’s threshold averaged across the remaining fitted conditions was used.

Figure 2A (left) shows the mean proportion “different” response averaged across participants as a function of condition and modulation depth difference. Twelve out of 29 participants met the performance criteria. As the Auditory-Only condition was run only on the first block, we analysed the auditory-only and auditory-visual data with separate repeated-measures analyses of variance (ANOVAs). Consistent with our predictions for the auditory-visual trials, the proportion “different” response was higher in the Sync-High (blue) compared to the Sync-Low (red) condition, *F*(1, 11) = 111.09, *p* < 0.001, ${\eta }_{p}^{2}=0.91$, but there was a significant interaction between pair type and modulation depth difference, *F*(4, 44) = 12.91, *p* < 0.001, ${\eta }_{p}^{2}=0.54$. Not surprisingly, the proportion “different” response increased as the modulation depth difference increased for auditory-only and auditory-visual trials (*p* < 0.001).

The presence of the cuboid on auditory-visual trials may lead to decisional biases rather than affecting perceptual discrimination. We therefore converted the proportion “different” response to a bias-free measure of sensitivity—the cumulative d’, which uses false alarms computed from the 0% modulation depth difference (i.e., “same” trials) for all other depth differences (i.e., “different” trials)^19^ (see Methods). Figure 2A (right) shows the mean cumulative d’ as a function of condition and modulation depth difference. Importantly, participants were more sensitive in the Sync-High (blue, *M* = 1.44, *SE* = 0.14) compared to the Sync-Low (red, *M* = 0.43, *SE* = 0.15) condition, *F*(1, 11) = 112.33, *p* < 0.001, ${\eta }_{p}^{2}=0.91$, and there was no significant interaction between pair type and modulation depth difference, *p* = 0.16. For auditory-only and auditory-visual trials, sensitivity increased as the modulation depth difference increased, *p*s < 0.001 (overall cumulative d’ for the Auditory-Only condition: *M* = 1.44, *SE* = 0.15).

To compare auditory-only and auditory-visual data, we conducted post-hoc *t*-tests and report Bonferroni-corrected *p*-values. First, these tests confirmed that cumulative d’ was lower in the Sync-Low compared to the Auditory-Only condition, *t*(11) = −5.92, *p* < 0.001, but not significantly different between the Sync-High and Auditory-Only conditions (see Supplementary Tables 1S–3S for comparison per modulation depth difference in this and subsequent experiments). Second, we compared the 75% threshold across the Auditory-Only, Sync-High and Sync-Low conditions (see Methods). Figure 2D shows the mean threshold averaged across participants as a function of condition. Consistent with the sensitivity results, for the same level of discrimination performance participants needed a larger modulation depth difference in the Sync-Low condition compared to the Auditory-Only, *t*(11) = 3.68, *p* = 0.01, or the Sync-High condition, *t*(11) = 3.97, *p* = 0.006. Thresholds also differed between the Auditory-Only and Sync-High condition, *t*(11) = 2.93, *p* = 0.04.

### Influence of vision on hearing depends on modulation synchrony

The results of Experiment 1 showed that task-irrelevant visual information strongly affected performance on an auditory discrimination task. To better understand the mechanisms of auditory-visual interactions, in Experiment 2 we tested the prediction that such interactions require auditory and visual changes to occur in synchrony^20,21^. Importantly our auditory and visual stimuli allowed us to manipulate synchrony equivalently in both modalities in terms of the modulation rate of amplitude or size changes. A second group of participants performed the same auditory discrimination task as in Experiment 1. They first completed one block of auditory-only trials, followed by two blocks of auditory-visual trials. In contrast to Experiment 1, on auditory-visual trials we also manipulated whether the cuboid’s size modulation was synchronous or asynchronous with the paired tone’s amplitude modulation. On synchronous trials the tone and cuboid were modulated at 2 Hz (Fig. 1B left), while on asynchronous trials the tone’s amplitude was modulated at 2 Hz and the cuboid’s size at 1 Hz (Fig. 1B right). There were thus five conditions in Experiment 2 (Auditory-Only; and Sync-High, Sync-Low, Async-High, and Async-Low from the factorial combination of synchrony and pair type).

Figure 2B shows the mean proportion “different” response and mean cumulative d’ as a function of condition and modulation depth difference. Nineteen out of 33 participants met the performance criteria. The auditory-visual data were submitted to a 2 × 2 × 5 ANOVA. Consistent with our prediction there was a significant 3-way interaction between synchrony, pair type and modulation depth difference for the proportion “different” response, *F*(4, 72) = 8.98, *p* < 0.001, ${\eta }_{p}^{2}=0.33$, and cumulative d’, *F*(3,54) = 3.19, *p* = 0.03, ${\eta }_{p}^{2}=0.15$. Post-hoc *t*-tests confirmed that when the modulation rate of the auditory and visual stimuli were synchronous, sensitivity showed a clear dependence on whether the cuboid was paired with the tone having the higher (Sync-High, blue, *M* = 1.32, *SE* = 0.17) or lower (Sync-Low, red, *M* = 0.48, *SE* = 0.15) modulation depth, *t*(18) = 6.75, *p* < 0.001 (compare Fig. 2A,B; for the Auditory-Only condition: *M* = 1.52, *SE* = 0.12). In comparison, when changes were asynchronous, there was no difference between the Async-High (cyan, *M* = 1.24, *SE* = 0.15) and Async-Low (orange, *M* = 1.12, *SE* = 0.14) conditions. There was a main effect of synchrony for cumulative d’; a main effect of pair type for both proportion “different” response and cumulative d’ (*p*s < 0.01); and pairwise interactions for both measures (*p*s < 0.02) except for the pair type × modulation depth difference for cumulative d’. In all conditions, both proportion “different” response and cumulative d’ increased with increasing modulation depth difference (*p*s < 0.001).

As in Experiment 1, we conducted post-hoc *t*-tests to compare auditory-only and auditory-visual data for cumulative d’ and threshold. These tests confirmed that cumulative d’ was higher in the Auditory-Only condition compared to the Sync-Low and Async-Low auditory-visual conditions (*p*s < 0.03), but not to the Sync-High and Async-High conditions. The threshold was significantly lower in the Auditory-Only condition compared to the Sync-Low condition, *t*(18) = −4.26, *p* < 0.001. Like sensitivity, thresholds differed between pair type with synchronous auditory and visual changes, *t*(18) = −4.00, *p* < 0.001, but not with asynchronous changes (Fig. 2D).

### Modulated sounds weakly influence the discrimination of modulated visual shapes

The results of the first two experiments demonstrated that participants’ performance on an auditory discrimination task was affected by a task-irrelevant visual stimulus, but only when it was synchronous with the auditory stimulus. In Experiment 3, we tested whether the reverse is also true; namely, does a task-irrelevant but synchronous auditory stimulus influence performance on a visual discrimination task? A third group of participants was presented with pairs of cuboids whose size modulated at 2 Hz, and judged whether the size modulation depth was the same or different. They first completed one block of visual-only trials, followed by one block of auditory-visual trials. On “different” trials one of the cuboids’ size had a fixed modulation depth of 36% and the other cuboid’s size had a modulation depth of either 40%, 44%, 48% or 52% i.e., modulation depth differences of 4% to 16% in 4% steps. This depth-difference range covered the same range of discriminability as the Auditory-Only condition in Experiments 1 and 2. On “same” trials, we randomly selected one of the modulation depths (36% to 52%) with replacement. On auditory-visual trials, an amplitude-modulated tone which had a modulation depth of 70% at 2 Hz was paired with either the cuboid with the higher (Sync-High) or lower (Sync-Low) size modulation depth. Thus, the auditory-visual discrepancy between the distractor modulation depth and maximum target modulation depth was the same across all three experiments (i.e., distractor modulation depth of 70% and maximum target modulation depth of 52%). No sound was presented with the other cuboid.

Figure 2C shows the mean proportion “different” response and cumulative d’ as a function of condition and difference in modulation depth. Twelve out of 17 participants met the performance criteria. There were important differences in the results between this experiment and Experiment 1, even though an analogous paradigm was used and the cumulative d’ in the single-modality condition did not significantly differ between the two groups. The proportion “different” response was not significantly different in the Sync-High compared to the Sync-Low condition, *F*(1, 11) = 3.62, *p* = 0.08, ${\eta }_{p}^{2}=0.25$, but there was a significant interaction between pair type and modulation depth difference, *F*(4, 44) = 3.19, *p* = 0.02, ${\eta }_{p}^{2}=0.23$. As with the auditory discrimination task in Experiments 1 and 2, the proportion “different” response increased as the modulation depth difference increased for visual-only and auditory-visual trials, *p*s < 0.001. Importantly for cumulative d’, participants were equally sensitive in the Sync-High (blue, *M* = 1.34, *SE* = 0.13) compared to the Sync-Low (red, *M* = 1.09, *SE* = 0.13) condition, *F*(1, 11) = 3.58, *p* = 0.09, ${\eta }_{p}^{2}=0.25$. There was also no interaction between pair type and modulation depth difference, *p* = 0.15. There was a main effect of modulation depth difference for visual-only and auditory-visual trials, *p*s < 0.001 (overall cumulative d’ for the Visual-Only condition: *M* = 1.17, *SE* = 0.15). Lastly, there were no significant differences for the 75% threshold between the Visual-Only, Sync-High and Sync-Low conditions, *p*s > 0.13, uncorrected (see Fig. 2D).

A comparison between Fig. 2A,C suggests that visual distractors (Experiment 1) had a larger effect on auditory discrimination than vice versa. To test this observation statistically, we first computed the difference in cumulative d’ between the Sync-High and Sync-Low condition at each depth difference for the auditory (Experiment 1) and visual (Experiment 3) discrimination task at each of the four stimulus levels; this difference reflects the size of the auditory-visual effect (i.e., the extent to which a distractor of the other modality influenced discrimination performance). We then submitted the data to a 2 × 4 mixed ANOVA. Importantly, there was a strong main effect of task group (i.e., experiment), *F*(1, 22) = 22.68, *p* < 0.001, ${\eta }_{p}^{2}=0.51$ (auditory task: *M*_*diff*_ = 1.02, *SE*_*diff*_ = 0.11; visual task: *M*_*diff*_ = 0.25, *SE*_*diff*_ = 0.11). That is, the auditory-visual effect (mean difference between the Sync-High and Sync-Low conditions) is more than four times larger for the group in which auditory discrimination was influenced by a visual distractor compared to the group in which visual discrimination was influenced by an auditory distractor. There was no main effect of stimulus level, *p* = 0.85, indicating that the auditory-visual effect was constant across all levels. There was an interaction between task group and stimulus level, *F*(3, 66) = 3.65, *p* = 0.02, ${\eta }_{p}^{2}=0.14$; post-hoc *t*-tests indicated that the difference between the two groups varied across stimulus levels (*p* < 0.001 to *p* < 0.08, uncorrected). Lastly, we submitted the auditory-visual threshold data to a 2 × 2 mixed ANOVA. Consistent with the cumulative d’ analyses, there were main effects of task group, *F*(1, 22) = 29.24, *p* < 0.001, ${\eta }_{p}^{2}=0.57$, and pair type, *F*(1, 22) = 16.76, *p* < 0.001, ${\eta }_{p}^{2}=0.43$, as well as a significant interaction between the two factors, *F*(1, 22) = 13.69, *p* = 0.001, ${\eta }_{p}^{2}=0.38$, with thresholds differing between the two auditory-visual conditions for the auditory-task group, but not for the visual-task group (as reported above). Thus, the presence of a synchronously modulated tone had a significantly smaller effect on the discrimination of the cuboid’s modulation depth. Importantly, participants’ cumulative d’ in the single cue Auditory-Only and Visual-Only conditions were matched across the three experiments. A 3 × 4 ANOVA revealed no main effect of experiment, *p* = 0.19, nor did experiment interact with stimulus level, *p* = 0.45. Participants further showed no statistical difference in their variance for the single-modality condition  across the three experiments (see Supplementary Table 4S). Overall, the results support the hypothesis that auditory-visual interactions occur between continuously modulated sounds and shapes, but vision has a much larger influence on hearing than hearing does on vision.

As noted above, many participants found the auditory discrimination task difficult and failed to meet our criteria for the Auditory-Visual condition. However, when we analysed separately those participants who failed to meet criteria, we found that while operating at a much lower level of performance overall, they demonstrated similar effects to those reported above (see Supplementary Figs 1S and 2S).

## Discussion

Our results show a clear auditory-visual interaction between equivalently modulated auditory and visual stimuli as long as both stimuli are modulated at the same rate, so that they change in a synchronised manner. Furthermore, using our novel modulation paradigm, we demonstrate that auditory-visual interaction is asymmetric: non-informative visual information influenced auditory discrimination (Experiments 1 and 2), but non-informative auditory information was significantly less effective in influencing visual discrimination (Experiment 3). Although in Experiments 1 and 2 pairing the shape synchronously with the least modulated sound impaired sensitivity relative to the Auditory-Only baseline, pairing the shape with the most modulated sound did not change sensitivity. It is not clear why there was no facilitation relative to baseline in this case. Our findings across Experiments 1 and 2 rule out the possibility that participants ignored the visual stimuli or that the results were due to learning effects or response biases.

We used different groups of participants for Experiments 1 and 3, but we manipulated the auditory and visual information equivalently and equated performance between groups for the Auditory-Only and Visual-Only conditions. Moreover, we ensured the modulation depth of task-irrelevant stimuli was easily detectable, but provided no information for the task in hand. Thus, even when auditory and visual modulation depths were matched such that there was no between-group difference in discriminability and variance between the two modalities, a strong unidirectional effect remained in which the visual information dominated.

Continuously modulated auditory and visual stimuli have previously been used to study auditory-visual interactions^20,21^, but not to determine whether each modality exerts equal influence on the other. For example, Maddox *et al*.^20^ used two streams of noise-modulated sound and a size-modulated visual disk to show that auditory selective attention to one sound stream is enhanced by the presence of a coherent visual stimulus, but there was no equivalent visual task in their study for comparison.

The question of modality dominance in auditory-visual interactions has received a good deal of attention in the literature^22^. It has been difficult to resolve because of the diversity of paradigms and stimuli used to address the nature of auditory-visual interactions and the problem of finding comparable tasks across modality. The modulations in our stimuli occur continuously a few times per second like many real-world stimuli. In contrast, auditory-visual studies often rely on the detection of non-overlapping transient stimuli only tens of milliseconds in duration that bear little resemblance to real-world objects^4,23,24^, or on stimuli in which auditory and visual properties conflict^8,25–27^. Some paradigms make it difficult to eliminate response bias from strictly sensory enhancement^28^, while others do not distinguish perceptual from attentional effects which can also modulate the integration of multisensory signals^29^. For instance, in paradigms to investigate the Colavita effect^4^, in which participants fail disproportionately to detect the auditory as opposed to the visual stimulus in a bimodal task^4,5,30–33^, visual dominance appears to arise from the visual capture of attention^5^.

Given these challenges, researchers have formalised modality dominance within a Bayesian framework contending that dominance depends on each modality’s reliability (i.e., inverse of variability) in estimating sensory information relevant for the task in hand^11,12,24,34,35^. Thus vision normally dominates (i.e., is weighted more) where the task requires a judgement of the spatial location of a stimulus (spatial task) such as in the ventriloquist effect^7,12,36–38^, but hearing has greater weight where the task requires judgement of a temporal property, such as stimulus presentation rate or interval judgement (temporal task)^8,9,24^. In support of this framework, the inherent superiority of a given modality for a task can be overcome by degrading information from that modality thereby decreasing its reliability which, in turn, reduces its relative weighting for that task^12^.

Our results, by comparison, show a dominance by vision using a discrimination task that did not inherently favour one modality over the other, and which, by avoiding stimulus degradation, allowed us to avoid any change to the appearance of our stimuli. Both auditory and visual stimuli were modulated signals that varied with time, but whose modulation rates were such that participants could readily follow the changes in either modality. In addition, we matched discriminability and variance of performance on the Auditory-Only and Visual-Only conditions between separate groups of participants in Experiments 1 and 3 (see Table 4S). We speculate, therefore, that under some circumstances, the interaction of auditory and visual information may not always follow Bayesian rules, but one modality, in this case vision, can dominate the other in the formation of a unified percept even when the reliability of the two modalities is equated. However, a more definitive test of the Bayesian hypothesis requires that reliability be equated for individual participants rather than at the group level as we have done here^11–13^.

Overall, our results suggest an important distinction between cross-modal *integration* and cross-modal *binding*^39^. The latter is a special case of integration occurring at a perceptual rather than a decisional stage and strongly requires some form of congruency of the information between different modalities. Given that we used dynamic stimuli, temporal coherence between the two modalities (i.e., synchrony) is the only way for establishing this congruence^39^. We conjecture that reliability may play a more prominent role at later integration stages. Like many auditory-visual signals associated with real-world objects, our auditory and visual stimuli are seconds in duration with congruent changes in amplitude and size. Consequently, they are more likely than transient flashes and sound bursts lasting only tens of milliseconds to be bound into a unified object. We speculate that our results suggest that the principles of integration for multimodal stimuli may depend on how readily two signals are perceptually bound, either as a result of any inherent dominance of one modality over the other or factors such as the temporal synchrony of the two signals.

## Methods

### Participants

Seventy-nine volunteers participated in the study. Twenty-nine participants (21 females, 8 males; age: *M* = 21 yrs, *SD* = 5 yrs), completed Experiment 1, thirty-three participants (21 females, 12 males; age: *M* = 24 yrs, *SD* = 3 yrs) completed Experiment 2, and seventeen participants (12 females, 5 males; age: *M* = 24 yrs, *SD* = 3 yrs) completed Experiment 3.

### Stimuli

#### Auditory stimuli

For the auditory discrimination task (Experiments 1 and 2), participants discriminated the modulation depth of amplitude-modulated tones 1500 ms in duration (Fig. 1A). A carrier frequency of 250 Hz was amplitude modulated at 2 Hz, with modulation depths that ranged from 20% to 52% in 8% steps. For the visual discrimination task (Experiment 3) the task-irrelevant sound used on auditory-visual trials was an amplitude-modulated tone with a single modulation depth of 70%. The rise and fall times of all tones were shaped with a 20-ms raised cosine function to avoid onset transients. Stimuli were generated in MATLAB 2012 (Mathworks, Inc.) with a sampling frequency of 44100 Hz and saved as .wav files for presentation. The sounds were presented to participants with Sennheiser HD380 Pro Headphones at a level of 65–70 dB SPL as measured using a Bruel and Kjaer Type 4152 Artificial Ear and a Type 2260 Hand Held Analyser.

#### Visual stimuli

For the visual discrimination task (Experiment 3) the stimuli were three-dimensional (3-D) cuboidal shapes. These were sinusoidally modulated in their girth at 2 Hz (Fig. 1B). The shapes were generated in 3D StudioMax 7 (Autodesk, Inc.) by applying the “spherify” modifier to the central portion of a blue cuboid (1.0 unit width × 1.2 unit height × 4.0 unit length). The value of this modifier can vary from 0% (rectangle) to 100% (sphere). As the value increased, the central portion became more spherical and therefore “bulged” outwards more than the end portions of the cuboid leading to size changes (see Fig. 1 and Videos). The values were modulated sinusoidally to generate cuboids with modulation depths that ranged from 36% to 52% in 4% steps. For the auditory discrimination task (Experiments 1 and 2), the task-irrelevant shape used on auditory-visual trials was the size-modulated cuboid but with a single modulation depth of 70%. The object was rendered against a uniform black background from an oblique camera viewpoint. The bounding box of the cuboid subtended a visual angle of 13.7° × 13.7° (300 pixels × 300 pixels). The videos were saved as Quicktime Movie Files (240 frames; 60 frames per second; H264 compression).

#### Auditory-visual stimuli

To create synchronous auditory-visual stimuli in all three experiments, both the tone and cuboid had a modulation rate of 2 Hz. To create asynchronous auditory-visual stimuli in Experiment 2, the tone had a modulation rate of 2 Hz whereas the cuboid had a modulation rate of 1 Hz (Compare Fig. 1B left and right).

### Design and procedure

Participants were tested individually in a dimly lit sound-attenuating room (IAC Acoustics). They were seated approximately 50 cm from the computer monitor. The Psychophysics Toolbox^37,38^ running under MATLAB was used to present the stimuli and control the experiments.

#### Auditory discrimination task (Experiments 1 and 2)

Participants discriminated the modulation depth of two sequentially presented amplitude-modulated tones. They completed one block of auditory-only trials first, followed by one or two blocks of auditory-visual trials.

In the Auditory-Only condition (Fig. 1C top), one interval contained a tone with a fixed modulation depth of 20%, while the other contained a tone with a modulation depth ranging from 20% to 52% in 8% steps for modulation depth differences of 0% (same trials) to 32% (different trials). The order of the intervals was randomised. On same trials, the modulation depth was randomly selected from the five possible depths with replacement. There were 16 trials at each depth difference for a total of 80 auditory-only trials.

On auditory-visual trials, the task-irrelevant cuboid was presented with the tone in one of the intervals. In Experiment 1, there were two randomly interleaved auditory-visual conditions (pair type), one in which the cuboid was presented with the tone with the higher modulation depth (Sync-High, Fig. 1C middle) and the other in which the cuboid was presented with the tone with the lower modulation depth (Sync-Low, Fig. 1C bottom). On same trials, the cuboid was randomly presented equally often in either interval. In Experiment 2, there were four randomly interleaved auditory-visual conditions from the factorial combination of synchrony and pair type. These include the two conditions as in Experiment 1 in which auditory and visual changes were synchronous (Sync-High and Sync-Low), and two analogous conditions in which the auditory and visual changes were asynchronous (Async-High and Async-Low). There were 16 trials of all conditions and modulation depth differences for a total of 160 trials in Experiment 1 run in a single block, and for a total of 320 trials in Experiment 2 run in two separate blocks.

#### Visual discrimination task (Experiment 3)

The design was analogous to that in Experiment 1, except that participants discriminated the modulation depth of two sequentially presented size-modulated cuboids. In the Visual-Only condition, one interval contained a cuboid with fixed modulation depth of 36%, while the other interval contained a cuboid with a modulation depth ranging from 36% to 52% in 4% steps for modulation depth differences of 0% to 16%. On auditory-visual trials, the task-irrelevant amplitude-modulated tone was presented with either the cuboid with the higher modulation depth (Sync-High) or the cuboid with the lower modulation depth (Sync-Low) as in Experiment 1. There were 16 repetitions of all conditions and modulation depth differences (80 visual-only trials and 160 auditory-visual trials).

Figure 1C illustrates the sequence of events for both the auditory and visual tasks using a hypothetical “different” trial in each condition in Experiment 1. Each trial began with a white fixation cross at the centre of the screen for 500 ms. A stimulus was presented for 1500 ms in the first interval and a second stimulus was presented for 1500 ms in the second interval. There was a 1000-ms blank interval in between the intervals. The trial ended with a green fixation dot in the centre of the screen which signalled the participants to respond. Participants used their left and right index finger to make a same or different response on a standard keyboard. The keys assigned to each response were counterbalanced across participants. Participants were instructed to respond as accurately as possible. They were further informed that an object (Experiments 1 and 2) or sound (Experiment 3) would also appear in one of the interval on each trial but that their task was only to decide whether or not the tone (or cuboid) in the two intervals had the same or different modulation depth. Prior to each block, participants were given 16 practice trials in which feedback (1000 Hz tone for 500 ms) was provided to ensure that they understood the task and to familiarize them with the trial sequence.

### Quantification and statistical analysis

We calculated each participant’s proportion “different” response from the number of “different” responses out of 16 repetitions for each condition and modulation depth difference. To calculate cumulative d’ for each participant we used the proportion “different” response at each non-zero modulation depth difference as the probability of a hit at each depth, and the proportion “different” response at 0% modulation depth difference as the probability of a false alarm for all non-zero depths^10^. Participants were only included in the final data analyses (Fig. 2) if they achieved a cumulative d’ of 1.5 (or greater) at the highest stimulus level on the Auditory-Only/Visual-Only condition. We additionally removed participants whose cumulative d’ was less than or equal to 0 for two or more of the remaining three stimulus levels (see Supplementary Data File). Analyses of the data of participants who failed to meet these criteria are shown in Figs 1S and 2S. The 75% threshold for each participant was calculated by fitting a cumulative Gaussian function to the proportion “different” response as a function of the modulation depth difference for each condition, and then estimating the modulation depth difference from the fitted curve that led to a 75% performance level. In cases in which a fit was not possible, we used the participant’s threshold averaged across the remaining fitted conditions. Repeated-measures ANOVA and *t*-tests in SPSS were used for all statistical analyses. For post-hoc comparisons we report Bonferroni corrected *p*-values (except where noted).

### Ethical approval and informed consent

The experimental protocols used in this study were approved by the Faculty of Medical Sciences Ethics Committee of Newcastle University. The research was conducted in accordance with the relevant institutional guidelines and regulations. All participants provided written informed consent, and were paid for their participation or received course credit.

## Supplementary information

Supplementary Information

Video 1

Video 2

Dataset 1

## Data Availability

The data generated during this study are included in this published article (see files in Supplementary Information).
