# Supplementary material for: Modulated stimuli demonstrate asymmetric interactions between hearing and vision
Source: Sci Rep. 2019 May 20;9:7605. doi: 10.1038/s41598-019-44079-5 (PMC6527605; doi:10.1038/s41598-019-44079-5)
Supplement: Supplementary file 1 — Supplementary Information [file 41598_2019_44079_MOESM1_ESM.pdf]

Modulated stimuli demonstrate asymmetric interactions between hearing and vision

Quoc C. Vuong, Mark Laing, Anjana Prabhu, Hei Iong Tung and Adrian Rees

Institute of Neuroscience, Newcastle University, Newcastle upon Tyne, NE2 4HH, UK

Supplementary Information

Video of synchronous (Video 1.mov) and asynchronous (Video 2.mov) bimodal stimuli

Examples of the auditory-visual stimuli used in the experiment. Video 1 is an example of a synchronous stimulus where both the tone and the shape were sinusoidally modulated at a rate of 2 Hz. Video 2 is an example of an asynchronous stimulus in which the sound is modulated at 2 Hz and the shape at 1 Hz. In both cases the tone is modulated at 52% the maximum depth used in the study, and the shape at 70% (other stimulus details as described in Methods).

Supplementary data file

Vuongetal_data.xlsx: This Excel file includes the proportion different, d prime, and parameter estimation data for all three experiments.

Supplementary tables and figures

Table 1S

Post-hoc t-tests of cumulative d' per modulation depth difference between the different conditions in Experiment 1. The t-value (df=11) is presented with the uncorrected p-value in parentheses.

	Modulation depth difference (%)			
	8	16	24	32
Sync-High vs Sync-Low	5.11 (<.001)	6.25 (<.001)	7.48 (<.001)	6.41 (<.001)
Auditory-Only vs Sync-High	-1.37 (.20)	.61 (.55)	-.19 (.86)	.77 (.46)
Auditory-Only vs Sync-Low	2.29 (.04)	5.34 (<.001)	6.28 (<.001)	7.23 (<.001)

Table 2S

Post-hoc t-tests of cumulative d’ per modulation depth difference between key conditions in Experiment 2. The t-value (df=18) is presented with the uncorrected p-value in parentheses.

		Modulation depth difference (%)			
		8	16	24	32
Sync	High vs Low	6.47 (<.001)	4.84 (<.001)	7.27 (<.001)	4.21 (.001)
	Auditory-Only vs High	.13 (.90)	.69 (.50)	1.25 (.23)	1.87 (.08)
	Auditory-Only vs Low	6.60 (<.001)	5.15 (<.001)	9.85 (<.001)	5.37 (<.001)
Async	High vs Low	2.70 (.015)	1.55 (.14)	-1.63 (.12)	.14 (.89)
	Auditory-Only vs High	1.13 (.27)	1.62 (.12)	1.92 (.07)	2.09 (.05)
	Auditory-Only vs Low	3.35 (.004)	2.32 (.03)	1.09 (.29)	2.65 (.02)

Table 3S

Post-hoc t-tests of cumulative d' per modulation depth difference between the different conditions in Experiment 3. The t-value (df=11) is presented with the uncorrected p-value in parentheses.

	Modulation depth difference (%)			
	4	8	12	16
Sync-High vs Sync-Low	1.88 (.09)	2.55 (.03)	.52 (.62)	.72 (.49)
Visual-Only vs Sync-High	-.01 (.99)	-1.72 (.11)	-1.12 (.29)	-.29 (.78)
Visual-Only vs Sync-Low	1.95 (.08)	.25 (.81)	-.89 (.39)	.42 (.69)

Table 4S

Variance estimated from individual subject's psychometric functions for the single-modality condition in each experiment.

	Mean	SEM
Expt 1 (E1): Auditory Discrimination	1.16	.09
Expt 2 (E2): Auditory Discrimination (Sync/Async)	1.19	.10
Expt3 (E3): Visual Discrimination	1.38	.09

Pairwise comparison	t-value	df	p-value
E1-E2	.23	29	.82
E1-E3	1.73	22	.10
E2-E3	1.31	29	.20

Figure 1S

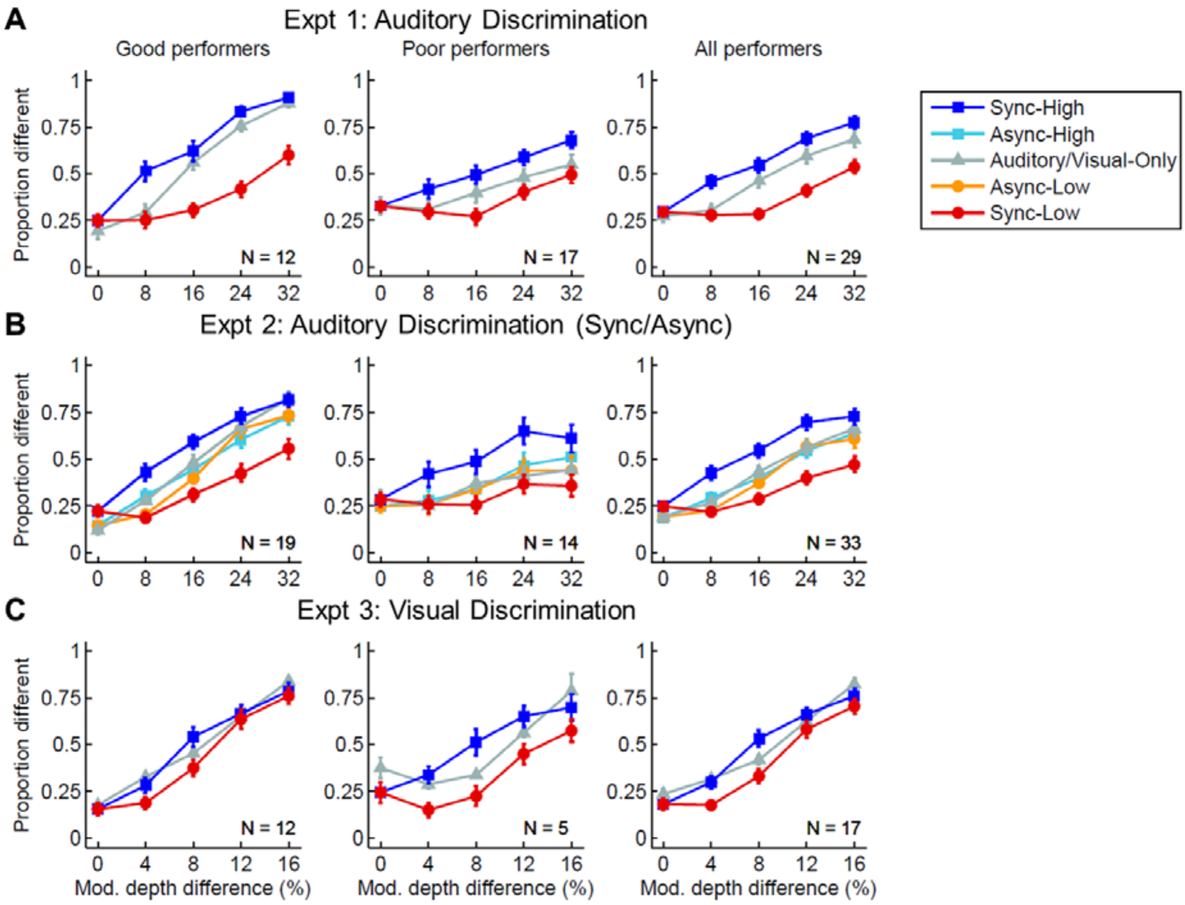

Figure 1S Caption

Proportion "different" data for good and poor performers (mean ± SE) in Experiments 1-3. Participants were categorised as good or poor performers depending on a performance criterion. Good performers had cumulative d' ≥ 1.5 at the largest modulation depth difference in the Auditory-Only or Visual-Only condition whereas poor performers did not reach this criterion. **A.** The mean proportion "different" response as a function of condition and modulation depth difference for good, poor and all performers in Experiment 1. The same data for good performers from Fig 2 are shown again for comparison. **B.** The mean proportion "different" response as a function of condition and modulation depth difference for good, poor and all performers in Experiment 2. **C.** The mean proportion "different" response as a function of condition and modulation depth difference for good, poor and all performers in Experiment 3.

Figure 2S

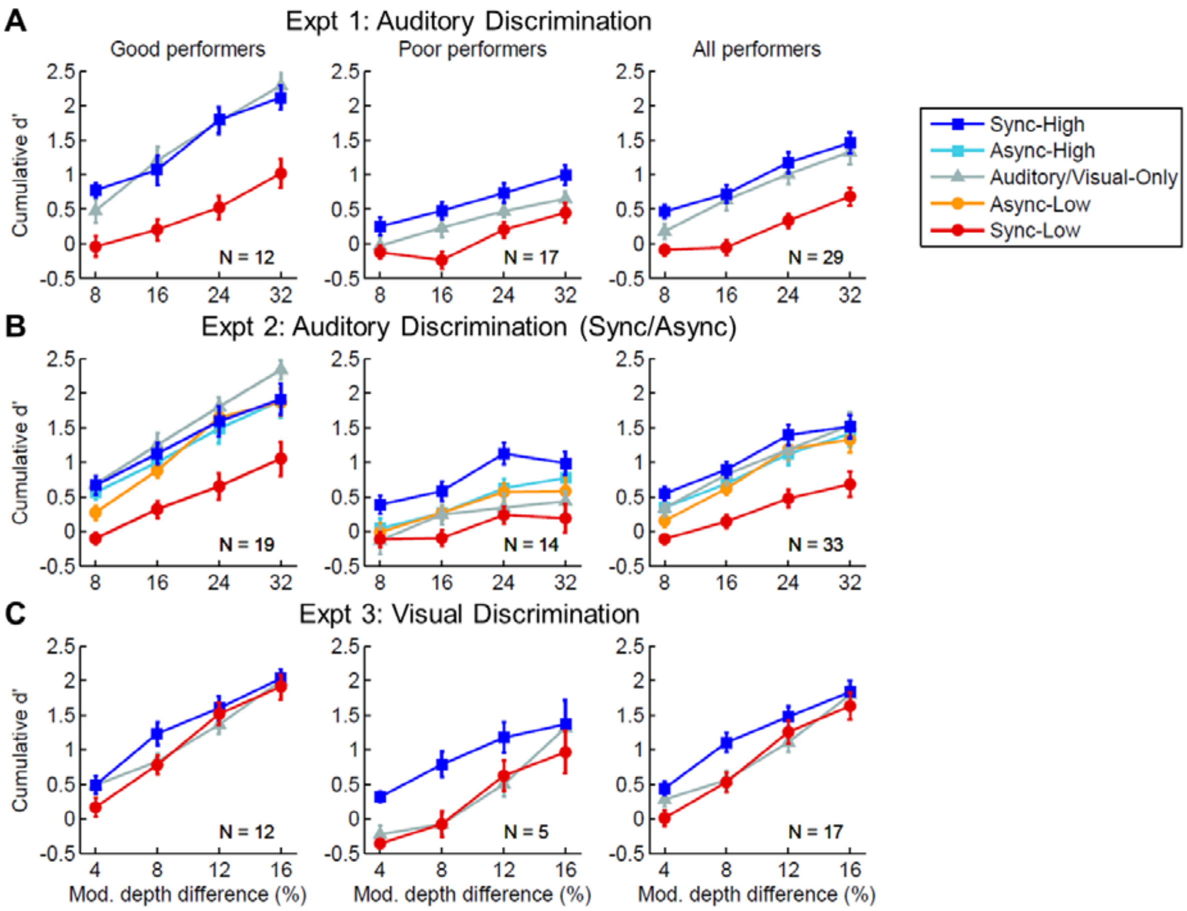

Figure 2S Caption

Cumulative d' data for good and poor performers (mean ± SE) in Experiments 1-3. **A.** The mean cumulative d' as a function of condition and modulation depth difference for good, poor and all performers in Experiment 1. **B.** The mean cumulative d' as a function of condition and modulation depth difference for good, poor and all performers in Experiment 2. **C.** The mean cumulative d' as a function of condition and modulation depth difference for good, poor and all performers in Experiment 3.
